# The severity and duration of Hypoglycemia affect platelet-derived protein responses in Caucasians

**DOI:** 10.1186/s12933-022-01639-w

**Published:** 2022-10-06

**Authors:** Abu Saleh Md Moin, Thozhukat Sathyapalan, Stephen L. Atkin, Alexandra E. Butler

**Affiliations:** 1 Research Department, Royal College of Surgeons in Ireland, PO Box 15503, Adliya, Bahrain; 2 Academic Endocrinology, Diabetes and Metabolism, Hull York Medical School, Hull, UK

**Keywords:** Type 2 diabetes, Hypoglycemia, Platelet-associated proteins, Inflammation

## Abstract

**Objective:**

Severe hypoglycemia is associated with increased cardiovascular death risk, and platelet responses to hypoglycemia (hypo) have been described. However, the impact of deep transient hypo (deep-hypo) versus prolonged milder hypo (mild-hypo) on platelet response is unclear.

**Research Design and methods:**

Two hypo studies were compared; firstly, mild-hypo in 18-subjects (10 type-2-diabetes (T2D), 8 controls), blood glucose to 2.8mmoL/L (50 mg/dL) for 1-hour; secondly deep-hypo in 46-subjects (23 T2D, 23 controls), blood glucose to < 2.2mmoL/L (< 40 mg/dL) transiently. Platelet-related protein (PRP) responses from baseline to after 1-hour of hypo (mild-hypo) or at deep-hypo were compared, and at 24-hours post-hypo. Slow Off-rate Modified Aptamer (SOMA)-scan plasma protein measurement was used to determine PRP changes for 13 PRPs.

**Results:**

In controls, from baseline to hypo, differences were seen for four PRPs, three showing increased %change in deep-hypo (Plasminogen activator inhibitor-1(PAI-1), CD40 ligand (CD40LG) and Protein-S), one showing increased %change in mild-hypo (von Willebrand factor (vWF)); at 24-hours in controls, %change for Protein-S remained increased in deep-hypo, whilst % change for vWF and plasminogen were increased in mild-hypo. In T2D, from baseline to hypo, differences were seen for 4 PRPs, three showing increased %change in deep-hypo (PAI-1, platelet glycoprotein VI and Tissue factor), one showing increased %change in mild-hypo (CD40LG); at 24-hours in T2D, %change for CD40LG remained increased, together with vWF, in deep-hypo.

**Conclusion:**

Both mild-hypo and deep-hypo showed marked PRP changes that continued up to 24-hours, showing that both the severity and duration of hypoglycemia are likely important and that any degree of hypoglycemia may be detrimental for increased cardiovascular risk events through PRP changes.

**Supplementary Information:**

The online version contains supplementary material available at 10.1186/s12933-022-01639-w.

## Introduction

Changes in platelet function have been shown following hypoglycemia in type 2 diabetes (T2D) that provoke platelet hyperactivity via impairing sensitivity to prostacyclin 24-hours following the hypoglycemic insult [1]. Others have shown that prothrombotic effects are persistent, lasting 7 days or more following a hypoglycemic event in individuals with T2D [2]. Induced hypoglycemia is associated with increased inflammatory and oxidative stress markers, and metabolic changes, in T2D subjects, and these markers remain elevated at 24-hours that may explain some of these platelet-associated changes [3; 4]. Similarly, studies in patients with type 1 diabetes and non-diabetic control subjects show hypoglycemia-induced platelet activation [5; 6] mediated via various mechanisms including an elevation in adrenaline, a potent counter-regulatory hormone [7; 8]. Changes in levels of cytokines and coagulation proteins, such as antithrombin decreases combined with prothrombotic protein increases, examples being factor VIII, D-dimer, fibrinogen and fibrin degeneration products, mediate the inflammatory response [9].

Patients with T2D have activated platelets that are increasingly known to be associated with the elevated prevalence of cardiovascular disease (CVD) in T2D [10]. Though critical for hemostasis, platelets are known to contribute to pathologies such as deep vein thrombosis, stroke and myocardial infarction [11]. Platelets mediate inflammation by releasing proinflammatory and prothrombotic molecules such as soluble CD40 ligand (CD40LG) [12]. CD40LG is part of the tumor necrosis factor superfamily, is mainly expressed by activated T/B cells and platelets, and is involved in the inflammatory response, exerting cytokine-like activity [13]. CD40LG is primarily produced by activated platelets [14], is pro-thrombotic as well as pro-inflammatory, and therefore is involved in the development of atherothrombosis and atherosclerosis [15]. Other critical components in the activated platelet response include plasminogen activator inhibitor-1 (PAI-1) (also known as serpin E1) is produced by various types of cells, such as endothelial and monocyte/macrophages, and is primarily stored in platelets; it functions as a major inhibitor of plasma fibrinolytic activity [16]. As an acute phase protein, it is closely linked with inflammatory cytokines, growth factors and hormones; by suppressing fibrinolysis, which results in fibrin deposition and, ultimately, tissue damage, its elevation represents a risk factor for thrombosis [17].

**Fig. 1:**
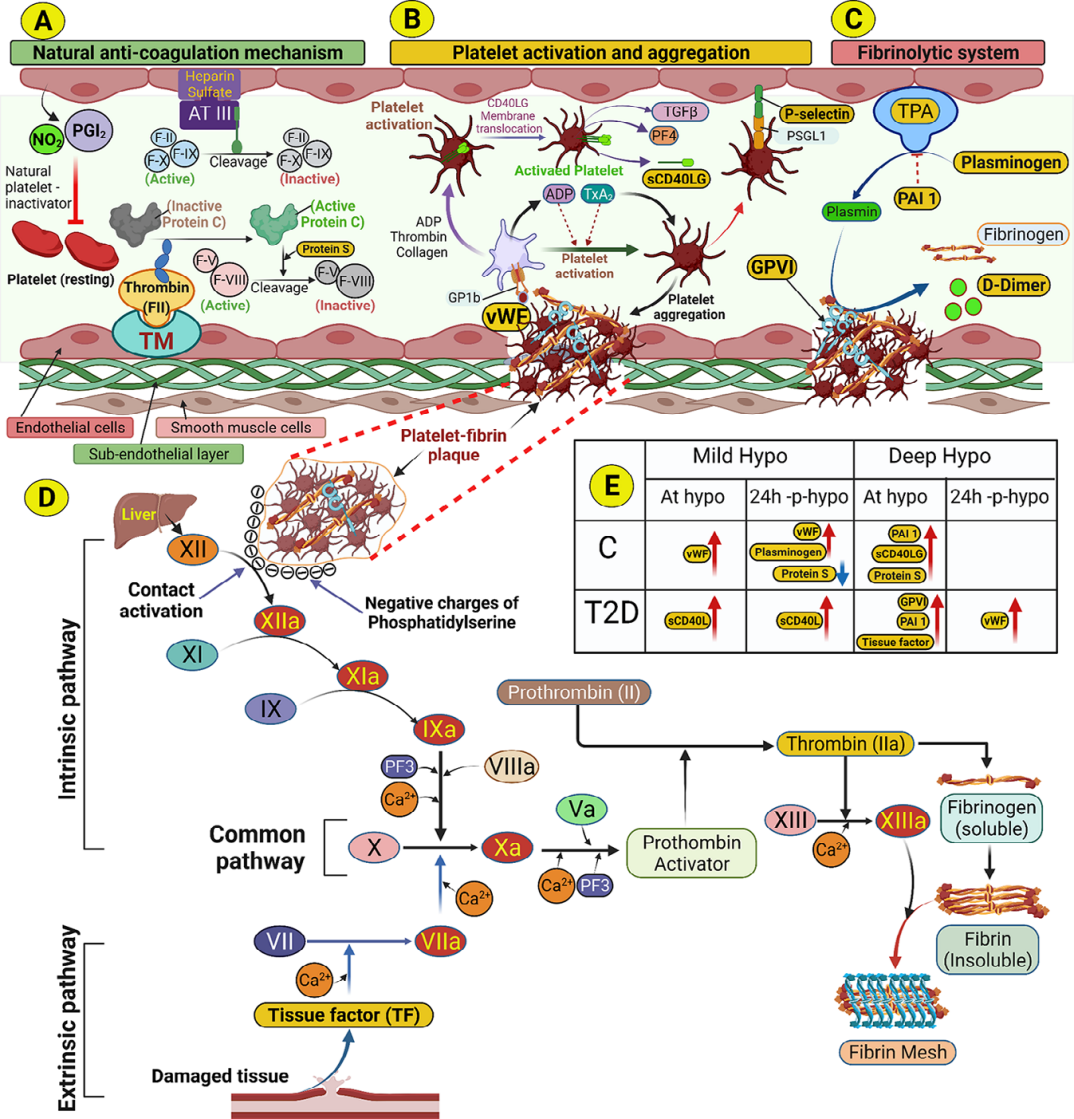
** A schematic illustrating the pathways and proteins involved in the natural mechanism that prevents blood from clotting, the activated platelet response and the degradation of fibrin clots. Created with Biorender.com ** **A.*Natural anti-coagulation mechanism***. In normal condition, nitric oxide (NO) and prostacyclin (PGI_2_) secreted from endothelial cells inhibit platelets, keeping them inactive and preventing them from binding to the endothelial lining. Platelet coagulation factors are also inactivated by antithrombin III (ATIII), a protein bound to endothelial cells by a glycose immune glycan (heparin sulfate) and activated protein C (APC) in association with protein S. ATIII cleaves circulating clotting factors (F-II, F-IX, F-X) and inactivates them. Protein C is activated by thrombin (F-II) bound to thrombomodulin (TM) in the endothelial cells. APCs cleaves circulating clotting factors (F-V, F-VIII) and inactivate them **B.*Platelet activation and aggregation.*** In response to damage to endothelial cells, circulating platelets migrate to the site of injury and bind to a protein, Von Willebrand factor (vWF), produced by endothelial cells through another platelet-surface protein glycoprotein 1b (GP1b) that activates the platelets. Activated platelets release granules containing adenosine di-phosphate (ADP) and thromboxane A2 (TXA2) which bind to their respective receptors expressed in platelets, allowing more platelets to migrate and form clusters at the site of injury, a process called ‘platelet aggregation’ through which ‘platelet-plaque’ is formed at the injury site. Platelet activation also allows the membrane translocation of CD40 ligand (CD40LG). The translocation of CD40LG seems to coincide with the release α-granule contents, including platelet-derived growth factor (PDGF), transforming growth factor beta (TGFβ) and platelet factor 4 (PF4). The surface-expressed CD40LG is cleaved and shed from the platelet surface in a time-dependent manner as sCD40LG. Platelet-endothelial interactions also promote progressive plaque, and this interaction is facilitated by rolling and adhering of activated platelets to the endothelial cell surface. Rolling of platelets to endothelial cells is mediated by platelet P-selectin glycoprotein ligand − 1 (PSGL-1) binding to endothelial cell P-selectin ***C. Fibrinolytic system.*** The final step of the blood homeostasis system involves the natural breakdown of the blood clot (platelet-fibrin plaque). Endothelial cells express a protein tissue plasminogen activator (TPA), which converts plasminogen to plasmin. Plasmin degrades the fibrin mesh in the platelet-fibrin plaque and releases fibrinogen and D-dimer. TPA is inhibited by an endothelial plasminogen activator inhibitor called plasminogen activator inhibitor-1 (PAI-1). ***D. Cascade pathways of blood coagulation system.*** The platelet surfaces in the platelet-fibrin clot (shown separately with broken, red-dotted lines) contain the phosphatidyl serine groups which are negatively charged. Coagulation factor XII, secreted from the liver, binds to the negatively charged surface and this contact triggers a conformational change in the XII zymogen, inducing autoactivation (contact activation). The activation leads the cascade pathway activation of XI, IX, VIII (intrinsic pathway). Tissue damage activates coagulation factors III and VII (extrinsic pathway) and, finally, both pathways activate factor X (common pathway) which leads to the conversion of prothrombin to thrombin. Thrombin converts fibrin polymer (insoluble) from fibrinogen monomers (soluble) and, with the help of XIII, fibrin finally forms a fibrin mesh to coagulate blood (with aggregated platelets at the site of injury) ***E. Comparison of % change of platelet activation-related proteins in type 2 diabetes.*** The table shows the % change of platelet activation-related proteins in response to mild and deep hypoglycemia in control subjects (C) and in subjects with type 2 diabetes (T2D). The upward red arrows indicate a % increase, and the downward blue arrow indicates a % decrease Hypo, hypoglycemia; 24 h-p-hypo, 24 h post-hypoglycemia; PF3, Platelet factor 3; C, control subjects; T2D, type 2 diabetes subjects

Platelet glycoprotein VI (GPVI) is a membrane glycoprotein that is unique to platelets and acts as a collagen receptor; thus, when vessel wall damage occurs, exposing subendothelial collagen, the collagen-GPVI interaction leads to activation of platelets, adhesion and thrombus formation [18; 19]. von Willebrand factor (vWF) is a large multimeric glycoprotein that serves as a key mediator of hemostasis via recruitment of platelets to the vessel wall injury site. However, it also promotes inflammation, and its “thrombo-inflammatory” characteristics are recognized as contributing to CVD [20]. Circulatory vitamin K-dependent protein S (protein S) is an essential anticoagulant, serving as a co-factor for activated protein C, that inactivate coagulation factors Va and VIIIa [21]. A small proportion of Protein S resides within the alpha granules of platelets, and may serve to counteract the procoagulant tendencies of platelets [22]. Tissue factor mediates hemostasis and is a thrombosis trigger, thereby playing a central role in atherothrombosis [23]. Tissue factor is predominantly expressed on circulating monocytes, though is also expressed on other cells, such as macrophages and platelets [24]. Plasminogen is a plasma protein that binds to platelets and to promote the generation of plasmin, an essential protein in fibrinolysis. In addition, plasminogen influences immune and inflammatory processes [25] and is implicated in a range of diseases, such as CVD, that have an inflammatory component [26]. D-dimer is a fibrinolytic product and increased levels have been used as a biomarker for thrombosis and thromboembolism [27]. However, it is also known to be an acute phase reactant, promoting inflammation by stimulating cytokines and interleukins, and is therefore a contributory factor to CVD [28] (Fig. 1).

It is unknown how the differing degrees of hypoglycemia may affect platelet related proteins (PRPs) that may then reflect changes in platelet function. We hypothesized that increasing the severity of hypoglycemia would have an incremental effect on any changes in PRPs; therefore, we determined levels of a panel of PRPs in plasma in patients with T2D and control subjects without diabetes in a study of mild prolonged hypoglycemia (mild-hypo) versus those changes in a study of deep transient hypoglycemia (deep-hypo) [1] [29].

## Research design and methods

### Study design

In both studies, a similar hypoglycemic clamp was employed that achieved the desired hypoglycemic level at 1 h in both controls and T2D.

### Mild-hypo design

A case-control prospective study in adult (aged 40–53 years) patients with T2D (n = 10) and nondiabetic control (n = 7) Caucasian subjects. A blood glucose of 2.8mmol/l (50 mg/dl) was achieved and then maintained for 1-hour [3]. Blood sampling was undertaken at baseline, at the end of 1-hour of hypoglycemia and at 24-hours post-hypoglycemia. Subjects did not have any overt symptoms of hypoglycemia,

This trial (mild-hypo) was approved by Yorkshire and the Humber Research Ethics Committee, registered at www.clinicaltrials.gov (NCT02205996) and performed from November 2011-May 2013.

### Deep-hypo design

A case-control prospective study was undertaken in 46 adult (aged 40–70 years) Caucasian subjects with T2D (n = 23) and non-diabetic controls (n = 23). A blood glucose of 2.0mmol/l (36 mg/dl) was achieved transiently and then reversed immediately [29]. Blood sampling was undertaken at baseline, at the point of hypoglycemia and at 24-hours post-hypoglycemia. All subjects developed sweating and became tremulous at hypoglycemia that were immediately reversed.

This trial (referred to here as deep-hypo) was approved by the North West-Greater Manchester East Research Ethics Committee, trial registration NCT03102801, and performed from March 2017-January 2018.

Both the mild-hypo and deep-hypo studies were undertaken in the Diabetes Centre at Hull Royal Infirmary. Written informed consent was provided by all participants.

### Study subjects

For patients with T2D, inclusion criteria in both studies included a diabetes duration < 10 years with stable dosage of medication (metformin, a statin and/or an angiotensin converting enzyme inhibitor/angiotensin receptor blocker) for at least the prior 3 months; metformin was the only anti-glycemic agent allowed; HbA1c levels < 10% (86mmol/mol)]; no hypoglycemic unawareness or hypoglycemia history during the prior 3-month period. Age was the only parameter that differed between the two groups with no difference in any of the other parameters including glycemic control or duration of diabetes (Table 1).

**Table 1:** Demographic and biochemical parameters of type 2 diabetic (T2D) subjects included in Mild-hypo and Deep-hypo. Data is presented as mean ± 1SD.

	Mild-hypo Type 2 Diabetes (n = 10)	Deep-hypo Type 2 Diabetes (n = 23)	P-value
Age (years)	46 ± 6	64 ± 8	< 0.0001
Sex (M/F)	7 M/3F	12 M/11F	
BMI (kg/m^2^)	36 ± 7	32 ± 4	0.051
Systolic BP (mmHg)	127 ± 20	132 ± 8	0.31
Diastolic BP (mmHg)	75 ± 11	81 ± 7	0.08
Duration of diabetes (years)	3.3 ± 2.3	4.5 ± 2.2	0.14
HbA1c (mmol/mol)	49 ± 12	51 ± 11	0.62
HbA1c (%)	6.6 ± 1.0	6.8 ± 1.0	0.48
Total cholesterol (mmol/l)	5.3 ± 0.7	4.2 ± 1.0	0.36
Triglyceride (mmol/l)	1.7 ± 0.8	1.7 ± 0.7	0.96
CRP (mg/l)	2.8 ± 1.8	3.1 ± 2.9	0.94

HbA1c = glycated hemoglobin; BMI = body mass index; CRP = C-reactive protein

For the control subjects, all underwent an oral glucose tolerance test to confirm non-diabetic status and the two groups differed in age alone (mild- hypo 47 ± 6 versus deep-hypo 60 ± 10 years) and did not differ in any of the measured parameters. All T2D and control participants had normal renal and hepatic function as assessed by biochemical indices, no history of cancer or any contraindication to hypoglycemia induction with insulin infusion. Medical history, clinical examination, routine blood tests and an electrocardiogram were performed on all participants.

### Biochemical markers

As previously described [3; 29; 30], “blood samples were separated immediately by centrifugation at 2000 g for 15 minutes at 4°C, and the aliquots were stored at − 80°C, within 30 minutes of blood collection, until batch analysis. Fasting plasma glucose (FPG), C-reactive protein (CRP), total cholesterol and triglycerides were measured enzymatically using a Beckman AU 5800 analyser (Beckman-Coulter, High Wycombe, UK)”.

### SOMA-scan assay

Thirteen platelet related proteins were measured using SOMAscan: PAI-1, CD40LG, protein S, vWF, plasminogen, D-dimer, GPVI, Tissue factor, platelet factor 4 (PF4), plasmin, P-selectin, prothrombin and fibrinogen gamma chain.

As previously described [30], “the SOMAscan assay used to quantify proteins was performed on an in-house Tecan Freedom EVO liquid handling system (Tecan Group, Maennedorf, Switzerland) utilizing buffers and SOMAmers from the SOMAscan HTS Assay 1.3K plasma kit (SomaLogic, Boulder, CO) according to manufacturer’s instructions and as described previously [31; 32].”

#### Data processing and analysis

As previously described [30], “Initial Relative Fluorescent Units (RFUs) were obtained from microarray intensity images using the Agilent Feature Extraction Software (Agilent, Santa Clara, CA). Raw RFUs were normalized and calibrated using the software pipeline provided by SomaLogic. This included (a) microarray hybridization normalization based on spiked-in hybridization controls, (b) plate-specific intensity normalization, (c) median signal normalization, and (d) median calibrator scaling of single RFU intensities according to calibrator reference values. Samples with a high degree of hemolysis (Haptoglobin log RFU < 10) were excluded from the analysis.

Statistical analyses were performed on log_2_ RFU values using R version 3.5.2 (R Foundation for Statistical Computing, Vienna, Austria) including base R package. Data handling and differential protein expression were analyzed using the autonomics and limma [33] packages. For differential protein analysis we applied limma models containing contrasts between timepoints, as well as contrasts between healthy and patients with diabetes at single timepoints. In both models, blocking by patient ID was performed to account for random effects. Batch effect correction was performed by adding batch as a covariate to the model. Limma obtained P values were corrected using the Benjamini-Hochberg method [34].”

### Statistical analysis

There are no studies to base a power calculation for platelet protein changes following hypoglycemia; however, based on our previous study to detect a significant change in platelet function following hypoglycemia measured as percentage inhibition in P-selectin expression in T2D, a sample size of 10 patients was calculated giving an effect size of 1.4 and 80% power to likely detect a change in platelet proteins with a alpha error of 0.05 [1]. Data trends were visually evaluated for each parameter and non-parametric tests were applied on data that violated the assumptions of normality when tested using the Kolmogorov-Smirnov Test. Comparison between groups was performed at each timepoint using Student’s t-test. Within-group comparisons of changes between timepoints were compared using Student’s t-test. Statistical analysis was undertaken using GraphPad Prism (San Diego, CA, USA).

## Results

Mild-hypo included 17 subjects [T2D (n = 10) and control (n = 7)] while deep-hypo included 46 subjects [T2D (n = 23) and control (n = 23)]. The demographic and biochemical data of the T2D participants in the mild-hypo and deep-hypo studies are summarized in Table 1. Participants did not differ with regard to their prescribed medication between studies.

Control (p = 0.003) and T2D (p < 0.0001) subjects in the deep-hypo study were older than their mild-hypo counterparts but did not differ for any other parameter including BMI, glycemic control in and diabetes duration in T2D or biochemical indices.

## Comparison of % change of protein levels in *control subjects* between study-1 and study-2

### Baseline to Hypoglycemia

**Fig. 2:**
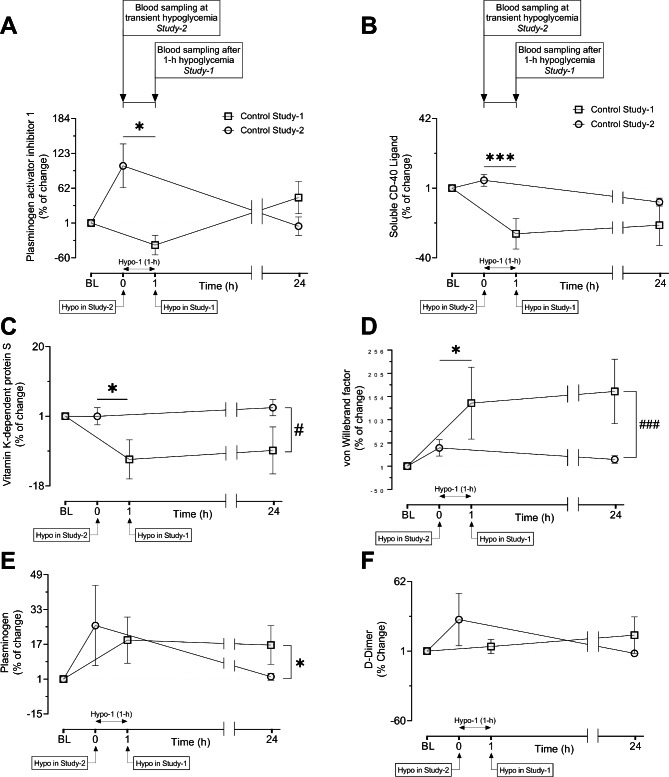
**Comparison of percent (%) changes of protein levels in response to hypoglycemia in two different prospective studies in CONTROL subjects.** Line graphs showing changes as percentage of six platelet activation related proteins, Plasminogen activator inhibitor 1 (PAI-1) (A) Soluble CD-40 ligand (B), Vitamin K-dependent protein S (C), Von Willebrand factor (D), Plasminogen (E), D-Dimer (F) from baseline (BL) to hypoglycemia and to 24 h post-hypoglycemia in study 1 (open white square) and study 2 (open white circle). Baseline protein levels were normalized to 1 to show the % change from baseline to subsequent timepoints. PAI-1 and soluble CD-40 ligand showed a significant differential percentage change from BL to hypoglycemia. Vitamin K-dependent protein S and Von Willebrand factor showed a significant differential percentage change from BL to hypoglycemia and from BL to 24 h post-hypoglycemia as well. Plasminogen and D-Dimer showed significant differential percentage change from BL to 24 h post-hypoglycemia only. Two-way arrows in the graphs indicate the duration of hypoglycemia for study 1. Data are presented here as mean % Change of proteins ± SEM. *p < 0.01, ***p < 0.001, % change of BL to hypo between study 2 vs. study 1 in control; #p < 0.05, ###p < 0.001, % change of BL to 24 h post-hypoglycemia between study 2 vs. study 1 in control. BL, baseline; Hypo, hypoglycemia

Percent change of 4 proteins (PAI-1, CD40LG, Protein S, vWF) differed in control subjects at baseline to hypoglycemia between mild-hypo (study-1) and deep-hypo (study-2); three of these proteins were higher in response to deep transient hypoglycemia (PAI-1, CD40LG, Protein S) and one (vWF) was higher in response to mild prolonged hypoglycemia in control subjects. Deep-hypo increased the %change of the following proteins in control subjects: PAI-1 (101.1 ± 38.4 vs. -37.5 ± 17.3%change of PAI-1 at hypoglycemia, study-2 vs. study-1, p = 0.03) (Fig. 2 A); CD40LG (5.7 ± 3.4 vs. -25.8 ± 9.0%change at hypoglycemia, study-2 vs. study-1, p = 0.0006) (Fig. 2B); protein S (1.0 ± 2.3 vs. -10.7 ± 5.3, %change at hypoglycemia, study-2 vs. study-1, p = 0.03) (Fig. 2 C). Mild prolonged hypoglycemia increased the %change of the following protein in control subjects: vWF (139.5 ± 79.0 vs. 41.2 ± 18.0%change at hypoglycemia, study-1 vs. study-2, p = 0.03) (Fig. 2D).

### Baseline to 24 h-posthypoglycemia

In control subjects, %change of vWF and plasminogen was greater at 24 h post-hypoglycemia in mild- hypo versus deep-hypo (165.1 ± 71.0 vs. 15.8 ± 8.1%change of vWF at 24 h-posthypo, study-1 vs. study-2, p = 0.001) (Fig. 2D); (16.6 ± 8.9 vs. 2.1 ± 1.5, %change at of plasminogen at 24 h post-hypoglycemia, study-1 vs. study-2, p = 0.013) (Fig. 2E). As at hypoglycemia, %change of protein S remained lower at 24 h post-hypoglycemia in mild-hypo versus deep-hypo (-8.3 ± 6.4 vs. 3.3 ± 2.2%change at 24 h post-hypoglycemia, study-1 vs. study-2, p = 0.03) (Fig. 2 C). The %change of CD40LG remained higher at 24 h post-hypoglycemia in deep-hypo; however, the level did not reach significance (-7.1 ± 2.3 vs. -20.7 ± 12.0%change of soluble CD-40 ligand, study-2 vs. study-1, p = 0.08) (Fig. 2 C). D-Dimer also appeared higher at 24 h post-hypoglycemia in control subjects in study-1 versus study-2 though, again, this did not reach significance (15.0 ± 16.0 vs. -0.9 ± 1.8%change of D-Dimer at 24-h post hypoglycemia, study-1 vs. study-2, p = 0.09) (Fig. 2 F).

## Comparison of % change of protein levels in *T2D subjects* between study-1 and study-2

### Baseline to Hypoglycemia

**Fig. 3:**
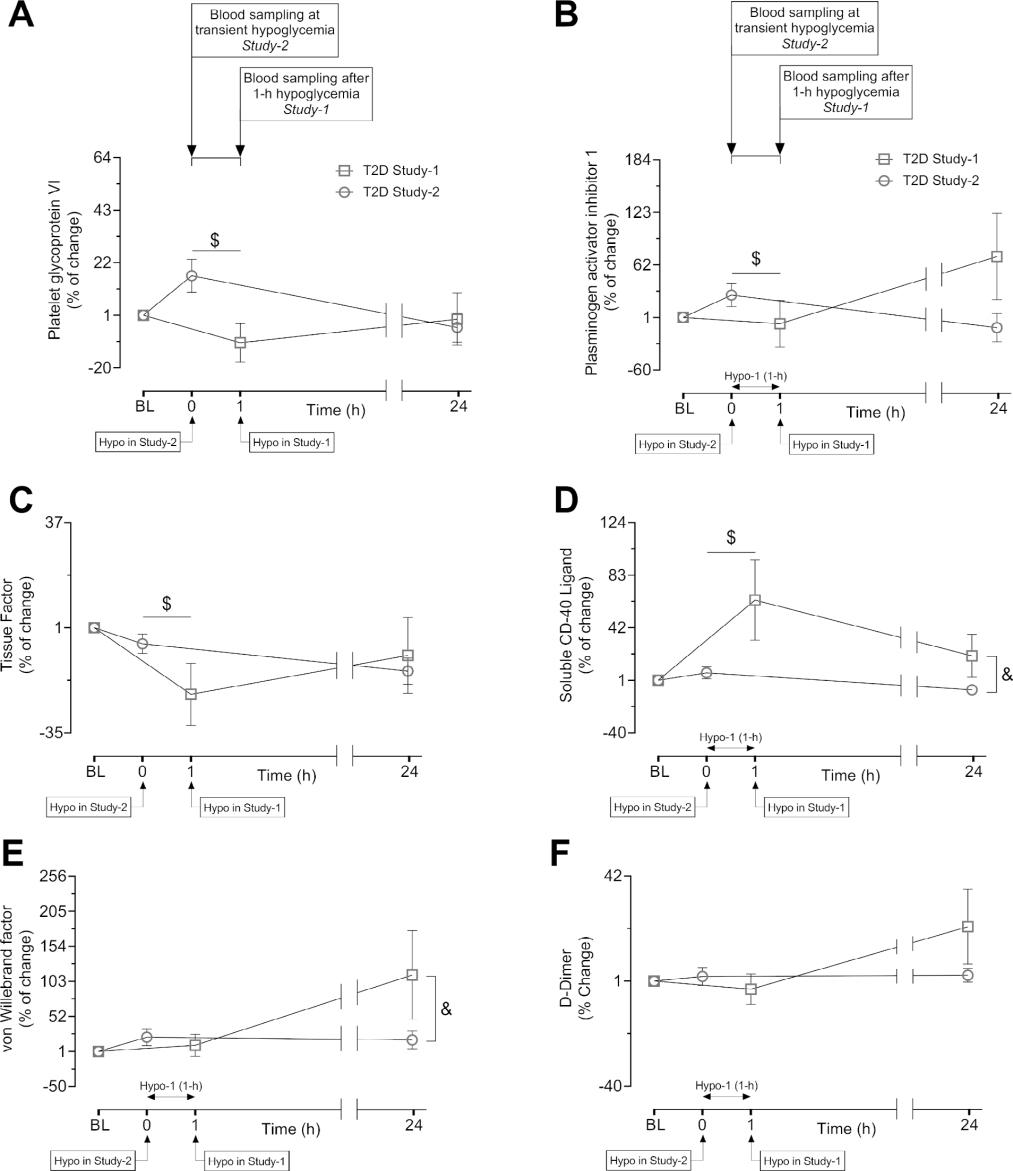
**Comparison of percent (%) changes of protein levels in response to hypoglycemia in two different prospective studies in T2D subjects.** Line graphs showing changes as percentage of six platelet activation related proteins, Platelet glycoprotein VI (A) Plasminogen activator inhibitor 1 (PAI-1) (B), Tissue factor (C), Soluble CD-40 ligand (D), Von Willebrand factor (E), D-Dimer (F) from baseline (BL) to hypoglycemia and to 24 h post-hypoglycemia in study 1 (open blue square) and study 2 (open blue circle). Baseline protein levels were normalized to 1 to show the % change from baseline to subsequent timepoints. Platelet glycoprotein VI and Plasminogen activator inhibitor 1 (PAI-1) showed a significant differential percentage change from BL to hypoglycemia. Tissue factor and Soluble CD-40 ligand showed a significant differential percentage change from BL to hypoglycemia and from BL to 24 h post-hypoglycemia as well. Von Willebrand factor and D-Dimer showed significant differential percentage change from BL to 24 h post-hypoglycemia only. Two-way arrows in the graphs indicate the duration of hypoglycemia for study 1. Data are presented here as mean % Change of proteins ± SEM. $p < 0.01, % change of BL to hypo between study 2 vs. study 1 in T2D; &p < 0.05, % change of BL to 24 h post-hypoglycemia between study 2 vs. study 1 in T2D. BL, baseline; Hypo, hypoglycemia

Percent change of 4 proteins (GPVI, PAI-1, tissue factor, CD40LG) differed in T2D subjects at baseline to hypoglycemia between deep-hypo (study-2) and mild-hypo (study-1); three proteins (GPVI, PAI-1, tissue factor) were higher in response to deep-hypo and one (CD40LG) was higher in response to mild-hypo in T2D subjects. Deep-hypo increased the %change of the following proteins in T2D: GPVI (16.8 ± 6.5 vs. -10.0 ± 7.7%change of GPVI at hypoglycemia, study-2 vs. study-1, p = 0.02) (Fig. 3 A); PAI-1 (27.0 ± 13.4 vs. -6.2 ± 27.1%change of PAI-1 at hypoglycemia, study-2 vs. study-1, p = 0.05) (Fig. 3B); Tissue factor (-4.5 ± 3.3 vs. -21.8 ± 10.61%change of Tissue factor at hypoglycemia, study-2 vs. study-1, p = 0.05) (Fig. 3 C). Mild-hypo increased the %change of CD40LG in T2D (49.3 ± 29.3 vs. 6.8 ± 4.6%change of CD40LG at hypoglycemia, study-1 vs. study-2, p = 0.014) (Fig. 3D).

### Baseline to 24 h post-hypoglycemia

%Change of CD40LG was higher at 24 h post-hypoglycemia in T2D in mild-hypo (20.0 ± 20.0 vs. -6.4 ± 2.8%change of CD40LG at 24 h post-hypoglycemia in T2D, study-1 vs. study-2, p = 0.033) (Fig. 3D). A similarly high %change of vWF was observed in mild-hypo compared to deep-hypo in T2D (112.3 ± 77.1 vs. 17.7 ± 13.0%change of vWF at 24 h post-hypoglycemia, study-1 vs. study-2, p = 0.05) (Fig. 3E). %Change of D-Dimer trended higher at 24 h in mild-hypo; however, the difference was not statistically significant (22.2 ± 17.4 vs. 3.2 ± 2.6%change of D-Dimer at 24 h, study-1 vs. study-2, p = 0.07).

None of the other PRPs differed between study-1 and study-2 at 24 h, suggesting that levels of these proteins had returned to baseline by 24 h post-hypoglycemia.

The %change of 2 proteins, PF4 and Plasmin, that tended to differ between studies at hypoglycemia in both control and T2D subjects but did not reach significance, is shown in Supplementary Fig. 1 A –B). Proteins that did not differ at any timepoints between deep-hypo and mild-hypo were P-Selectin, prothrombin and fibrinogen gamma chain (Supplementary Fig. 1 C–E).

Glucagon as a measure of the counter regulatory response was measured and its appropriate response in T2D and controls following hypoglycemia is shown in Supplementary Fig. 2.

## Conclusion

This is the first study to compare platelet-related protein responses to differing severity and length of hypoglycemia. Here, we show that in the control subjects that platelet-related proteins (PRPs) responded to hypoglycemia, with changes in both mild-hypo and deep-hypo that differed, but with residual changes at 24 h indicating that the response differs according to the degree and perhaps the duration of the event. Changes in PRPs were also seen in T2D, though that differed from controls suggesting that even in diabetes of shorter duration that the hypoglycemic response is modified. From baseline to hypoglycemia, %change differences between studies were seen in PRPs for both T2D (PAI-1, CD40LG, GPVI, tissue factor) and controls (PAI-1, CD40LG, Protein S, vWF); two proteins being common (PAI-1, CD40LG). PAI-1 showed a similar increase in %change in deep-hypo in both T2D and controls between studies whilst, interestingly, CD40LG showed an increase %change in deep-hypo in controls between studies but, conversely, showed increase %change in mild-hypo in T2D. The additional protein showing increased %change in controls in response to deep-hypo compared to mild-hypo was Protein S, while vWF showed an increase in %change in response to mild-hypo. In T2D, the additional differentially changed proteins, GPVI and Tissue factor, both showed increased %change in response to deep-hypo compared to mild-hypo.

By 24-hours, in controls, Protein S continued to show increased %change in response to deep-hypo and vWF continued to show increased %change in mild-hypo; plasminogen emerged at this timepoint as showing increased %change in milder prolonged hypoglycemia. By 24-hours, in T2D, CD40LG continued to show increased %change in milder prolonged hypoglycemia; vWF emerged at this timepoint similarly showing increased %change in milder prolonged hypoglycemia. At 24-hours, D-dimer also showed a trend toward increase in milder prolonged hypoglycemia in both T2D and controls, though this did not reach significance in either cohort.

The mechanism by which hypoglycemia may affect platelet proteins is complex and multifactorial. Glucagon, the principal counterregulatory hormone in response to hypoglycemia, has been reported to increase platelet aggregation in humans [35]. Our data also demonstrated elevated levels of glucagon in response to hypoglycemia in control subjects as well as subjects with T2D. Furthermore, induction of hypoglycemia in humans involves significant changes of physiological pathways. For example, in response to hypoglycemia, glucose counterregulatory mechanisms (involving sympathoadrenal system) are activated. The sympathoadrenal response includes activation of the adrenal medulla to secrete epinephrine and norepinephrine as well as activation of the sympathetic nervous system to release norepinephrine and acetylcholine. Epinephrine and norepinephrine act on the liver via β-2 adrenergic receptors to increase hepatic glycogenolysis and gluconeogenesis [36] to counteract hypoglycemia. Previous reports have demonstrated that glucose counterregulatory hormones are key mediators of platelet activation proteins. Epinephrine interacted with α2-adrenargic receptor on human platelets and potentiated the biochemical and aggregatory responses [37]. Epinephrine has also been reported to induce the release of other platelet activation factors, such as von Willebrand factor (vWF) from cultured endothelial cells [38], tissue type plasminogen activator (tPA) in human [39] and from perfused rat hindlegs [40] or in aged rats [41]. Therefore, it is likely that hypoglycemia-induced epinephrine might control the levels of platelet activation proteins in our study and we have reported changes in epinephrine in response to hypoglycemia previously [3].

Study strengths were the use of a similar hypoglycemic clamp to achieve the desired hypoglycemic level within the same time frame, both groups were well matched other than for age and the inclusion of patients with T2D with a relatively short disease duration. Small study numbers are a limitation, as with more participants, greater differences in PRPs may have become apparent and a type 2 statistical error cannot be excluded especially in the control population. It cannot be excluded that changes seen in the PRPs may have been due to the rate to achieve hypoglycemia in each study rather than the absolute levels of hypoglycemia achieved and this needs to be addressed in the future. A further limitation is that all subjects were Caucasian and the findings reported here may not be reflected in other ethnic populations.

Severe hypoglycaemia is associated with major adverse cardiovascular events (MACE) and all-cause mortality across diabetes landmark trials such as ACCORD, ADVANCE, ORIGIN and DEVOTE [42; 43; 44; 45] with the severity of hypoglycemia being more widely reported; however, in the EXAMINE study, both serious and any hypoglycaemia was associated with increased risk of MACE [46]. The study here suggests that, in addition to the severity of a hypoglycemic event, that the temporal duration is important and that those patients with tight control, but with multiple mild and frequent hypoglycemia are also at increased cardiovascular risk contributed to by changes in platelet parameters.

What can be seen from these results is that the pattern of PRP changes differed with the degree of hypoglycemia and, also, that the PRP changes differed between control and T2D for both mild prolonged and deep transient hypoglycemia. Given that the majority of the PRPs are involved in the inflammatory process, the chronic inflammation characteristic of T2D may have modified the PRP response, causing the differences seen here in comparison with controls. Hypothetically, increasing age and BMI, together with development of diabetes, diabetes duration and glycemic control, may further enhance the inflammatory response following hypoglycemia and thereby predispose an individual to a CVD event.

Overall, these changes may suggest that inflammatory pathways were activated by both hypoglycemic insults in T2D, and by a greater degree at the point of hypoglycemia in deep transient versus mild prolonged. Following hypoglycemia, changes in some PRPs persisted to 24-hours, suggesting that the increased risk from a significant hypoglycemic event wanes gradually over a period of at least 24-hours. It has been well documented that platelet changes may last 24-hours and beyond; however, what this study shows is that both deep transient and mild prolonged hypoglycemic events can effect changes in platelet proteins out to, and likely beyond, 24-hours, but that more changes were induced at 24-hours by milder, prolonged hypoglycemia.

In conclusion, both mild-hypo and deep transient hypoglycemia showed marked platelet protein changes that continued up to 24 h showing that both the severity and duration of hypoglycemia are likely important and that any degree of hypoglycemia may be detrimental for increased cardiovascular risk events through platelet protein changes.

## Electronic supplementary material

Below is the link to the electronic supplementary material.

Supplementary Material 1

Supplementary Material 2

Supplementary Material 3

## Data Availability

All the data for this study will be made available upon reasonable request to the corresponding author.
