# Supplementary figures and images for: The severity and duration of Hypoglycemia affect platelet-derived protein responses in Caucasians
Source: Cardiovasc Diabetol. 2022 Oct 6;21:202. doi: 10.1186/s12933-022-01639-w (PMC9541052; doi:10.1186/s12933-022-01639-w)

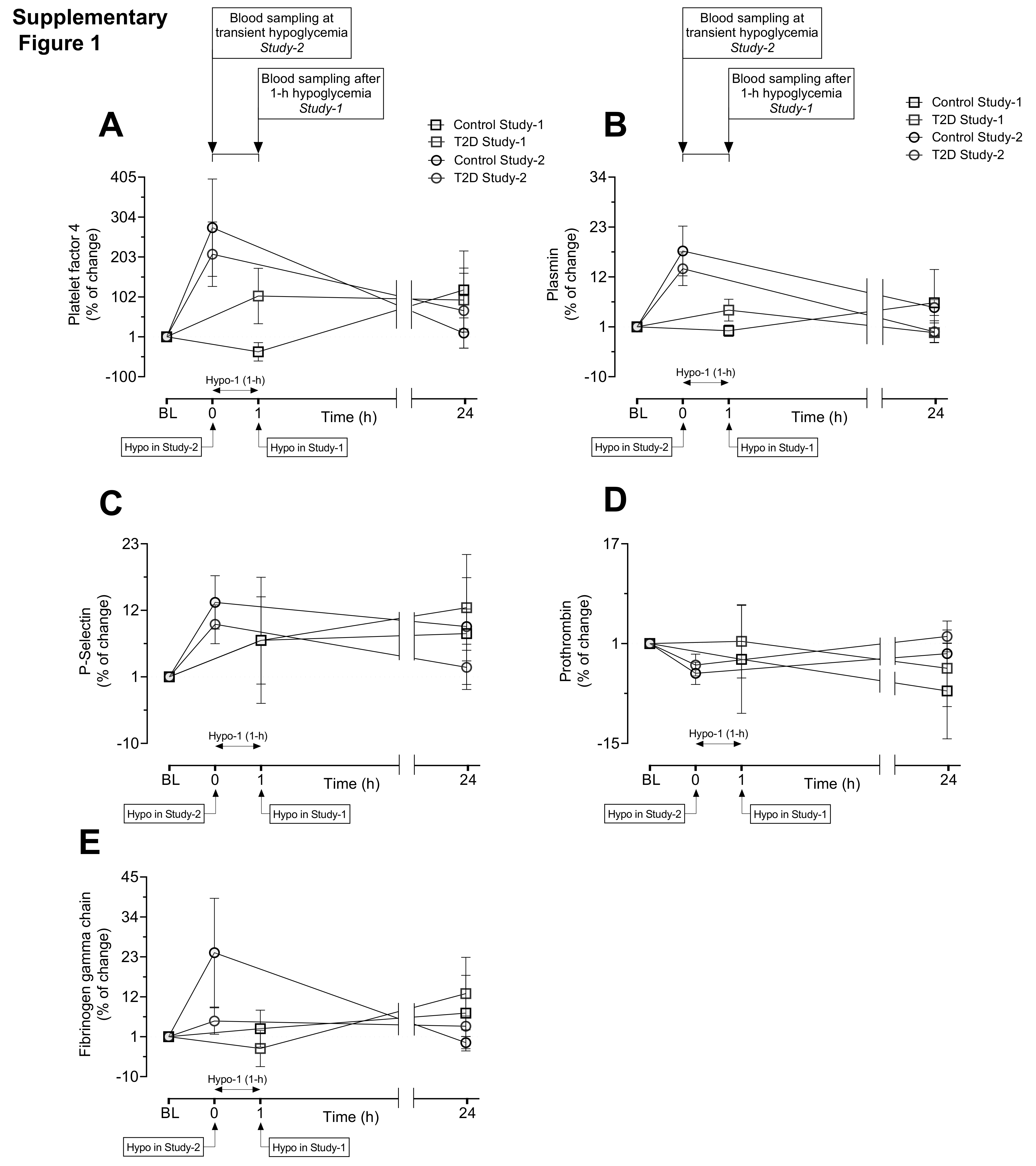

Supplement: Supplementary file 1 — Supplementary Material 1 [file 12933_2022_1639_MOESM1_ESM.tif]

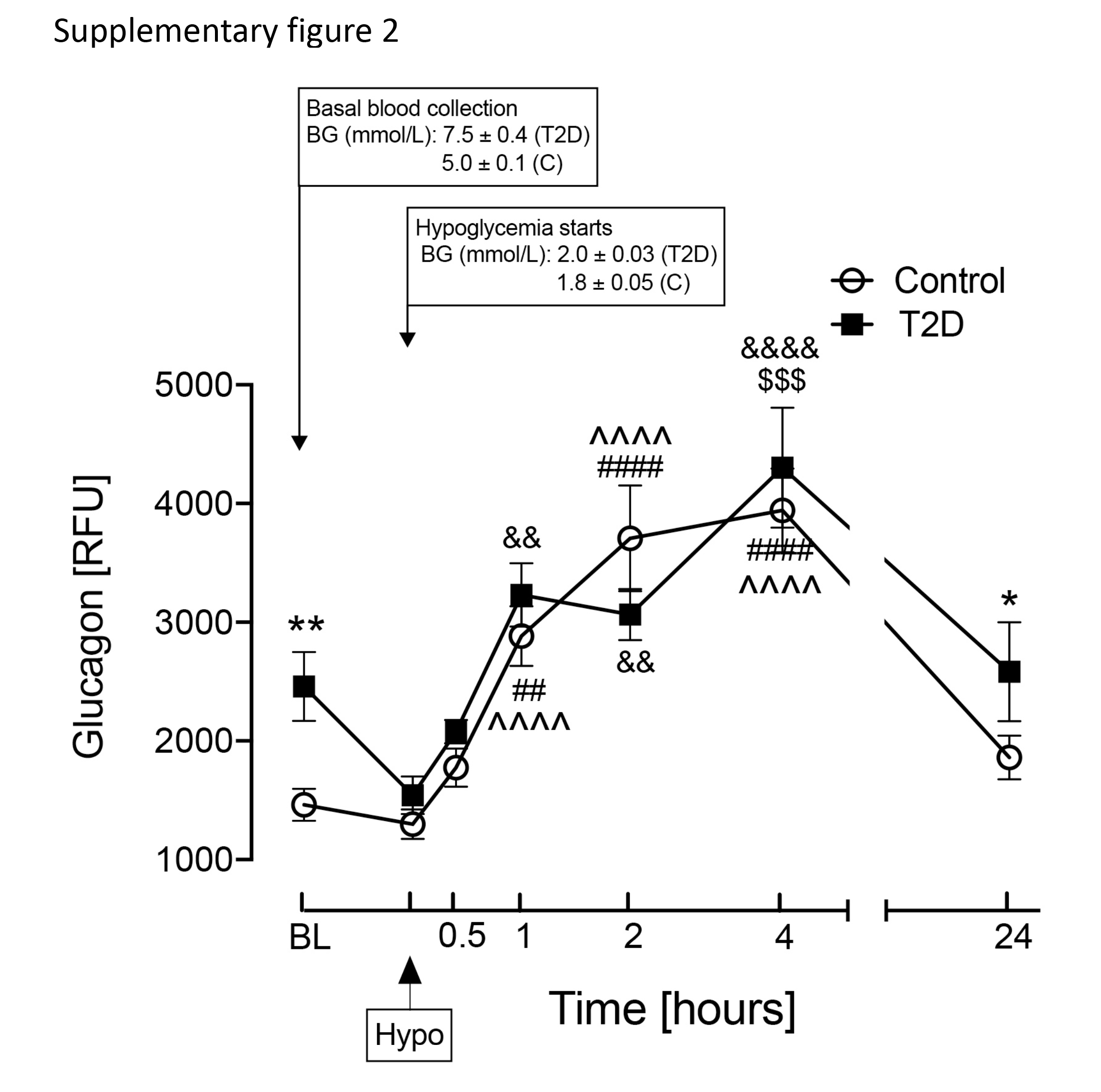

Supplement: Supplementary file 2 — Supplementary Material 2 [file 12933_2022_1639_MOESM2_ESM.tif]
