# Supplementary material for: The severity and duration of Hypoglycemia affect platelet-derived protein responses in Caucasians
Source: Cardiovasc Diabetol. 2022 Oct 6;21:202. doi: 10.1186/s12933-022-01639-w (PMC9541052; doi:10.1186/s12933-022-01639-w)
Supplement: Supplementary file 3 — Supplementary Material 3 [file 12933_2022_1639_MOESM3_ESM.doc]

**Supplementary Figure 1:** **Comparison of percent (%) changes of protein levels in response to hypoglycemia in two different prospective studies in control and T2D subjects.** Line graphs showing changes as percentage of five platelet activation related proteins that did not differ in any timepoints between study 1 and study 2. % Change of Platelet factor 4 (A), Plasmin (B), P-selectin (C), Prothrombin (D), Fibrinogen gamma chain (E) from baseline (BL) to hypoglycemia and to 24 hours post-hypoglycemia in study 1 (open white square, control, and open blue square, T2D) and study 2 (open white circle, control, and open blue circle, T2D). Baseline protein levels were normalized to 1 to show the % change from baseline to subsequent timepoints. Two-way arrows in the graphs indicate the duration of hypoglycemia (1-hour) for Mild-hypo. Data are presented here as mean % change of proteins ± SEM. BL, baseline; Hypo, hypoglycemia; 1-h, 1-hour.

**Supplementary Figure 2: Glucagon response throughout the experimental time course for the control and type 2 diabetes [T2D] cohorts.** Data is presented as mean ± SEM.

Statistics: Changes in protein levels within group (control and T2D) at all timepoints were compared using ANOVA. T2D, baseline to subsequent timepoints: $$$ p<0.001; T2D, hypoglycemia to subsequent timepoints: && p<0.01, &&&& p<0.0001; Control, baseline to subsequent timepoints: #### p<0.0001; Control, hypoglycemia to subsequent timepoints: ^^^^ p<0.0001.

RFU-relative fluorescent units; Hypo-hypoglycemia
